# Supplementary material for: A virophage cross-species infection through mutant selection represses giant virus propagation, promoting host cell survival
Source: Commun Biol. 2020 May 21;3:248. doi: 10.1038/s42003-020-0970-9 (PMC7242381; doi:10.1038/s42003-020-0970-9)
Supplement: Supplementary file 1 — Supplementary Information [file 42003_2020_970_MOESM1_ESM.pdf]

Supplementary Figures

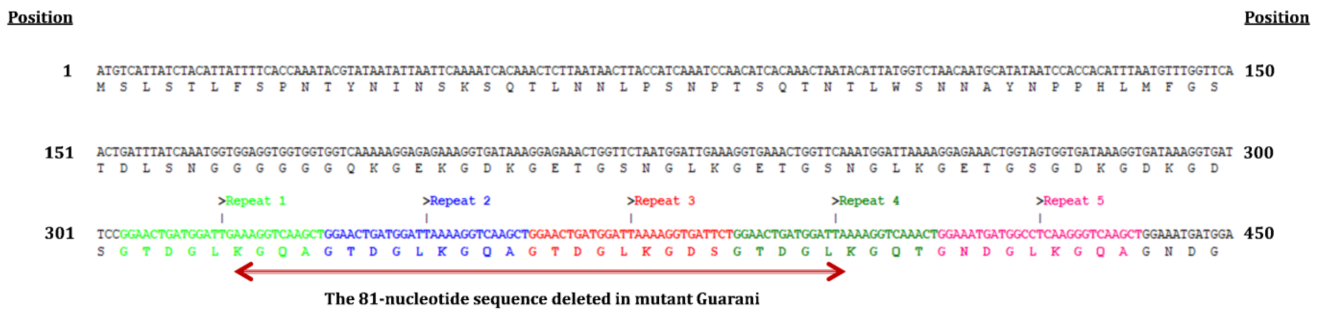

Supplementary Figure 1: Location of the deletion in the collagen-like gene of Guarani. The collagen-like repeats and their amino acid motifs are highlighted in colors. Only a part of the gene is represented here.

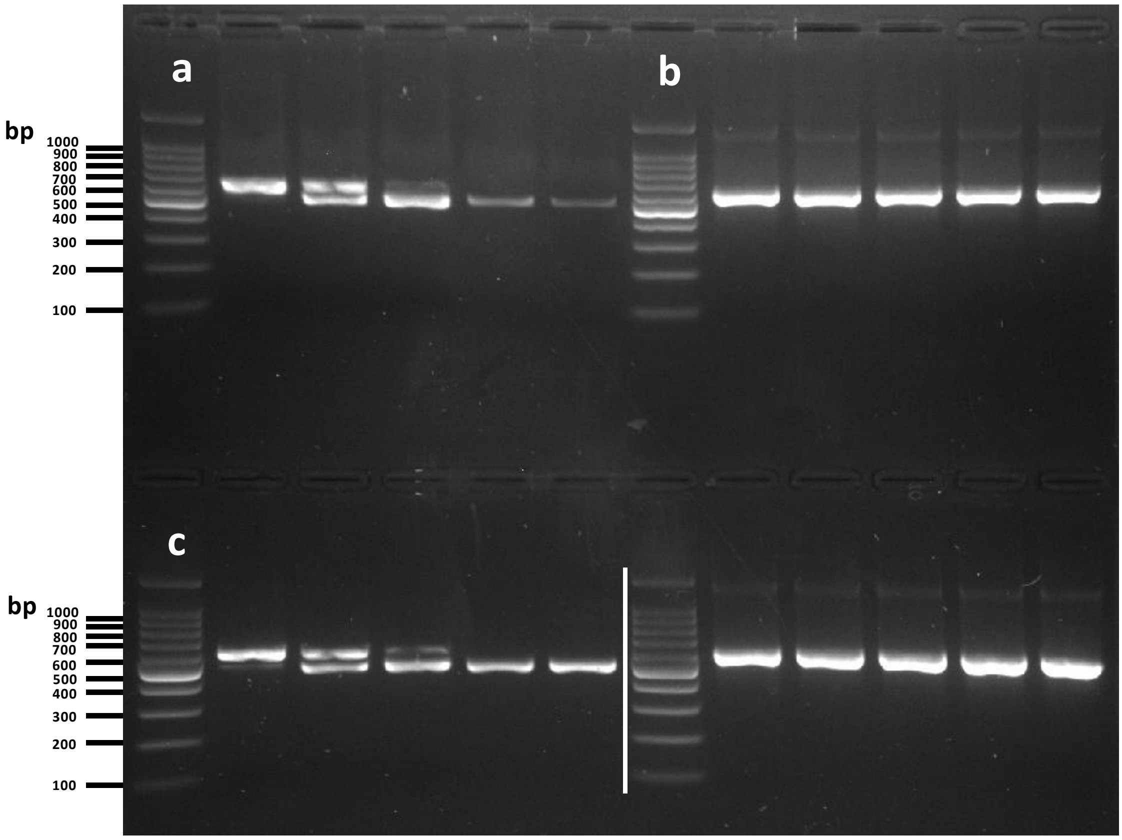

Supplementary Figure 2: original gel of Figure 3a - 3c. (**a**) Selection of the Guarani mutant genotype coinfecting Tupanvirus during a 5-passage experiment. (**b**) Maintenance of wild-type Guarani through passages with APMV (control). (**c**) The same experiment in **a** was repeated by adding fresh Tupanvirus at MOI of 10 at each passage.

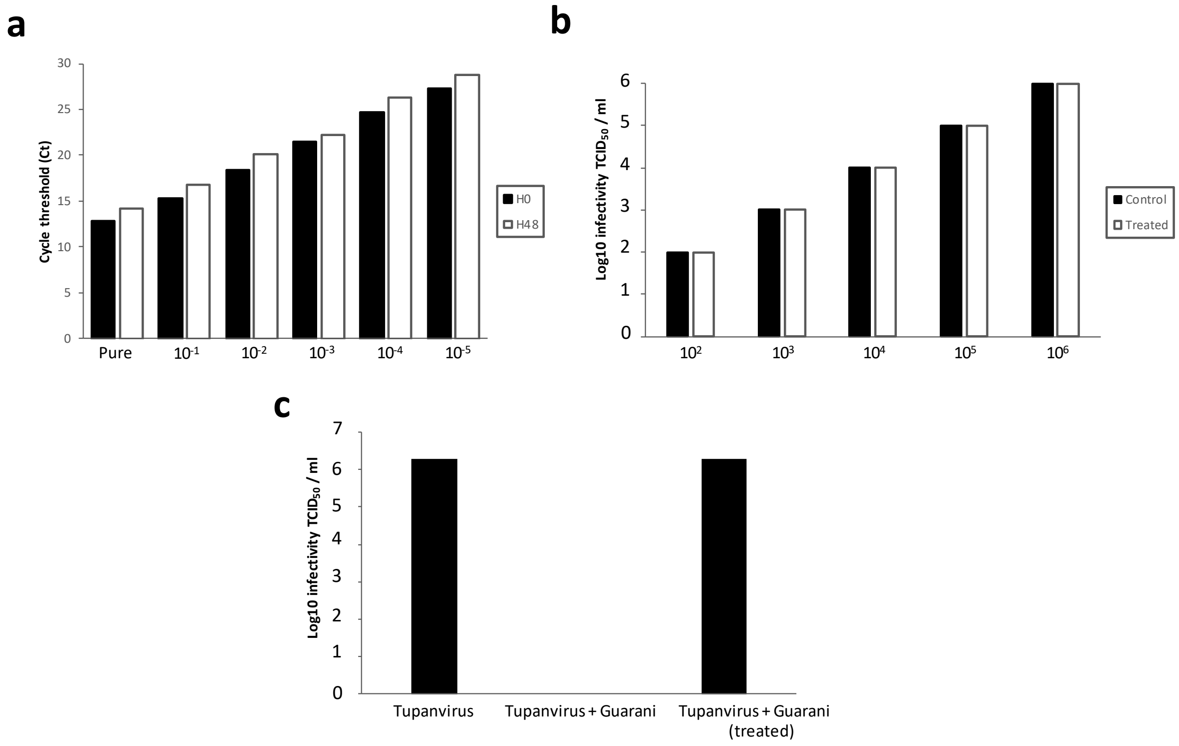

Supplementary Figure 3: Selective inactivation of virophage virions contained in the mixture wild-type and mutant Guarani using heat treatment at 55 °C for 30 min. (**a**) The supernatant collected from passage 2 with Tupanvirus was filtered through a 0.22-µm-pore filter to remove giant virus particles. The obtained supernatant was then serially diluted from 10^{-1} to 10^{-5}. Each dilution was then submitted to heat treatment and subsequently used to coinfect *A. castellanii* cells simultaneously inoculated with Tupanvirus Deep Ocean. The virophage did not replicate in all conditions, indicating that heat treatment successively inactivated it. (**b**) Different concentrations of Tupanvirus Deep Ocean were submitted to heat treatment and then titrated using end point dilution. We did not observe any change in the titer of viable particles after the treatment. (**c**) *A. castellanii* cells were inoculated with Tupanvirus at an MOI of 1 and Guarani mixture at an MOI of 10. Prior to coinfection, virus-virophage

suspensions were mixed, incubated to allow composite formation and then submitted to heat treatment. After lysis, the titer of infectious particles was measured by end point dilution. Heat treatment completely prevented virophage inhibition and allowed Tupanvirus to replicate.

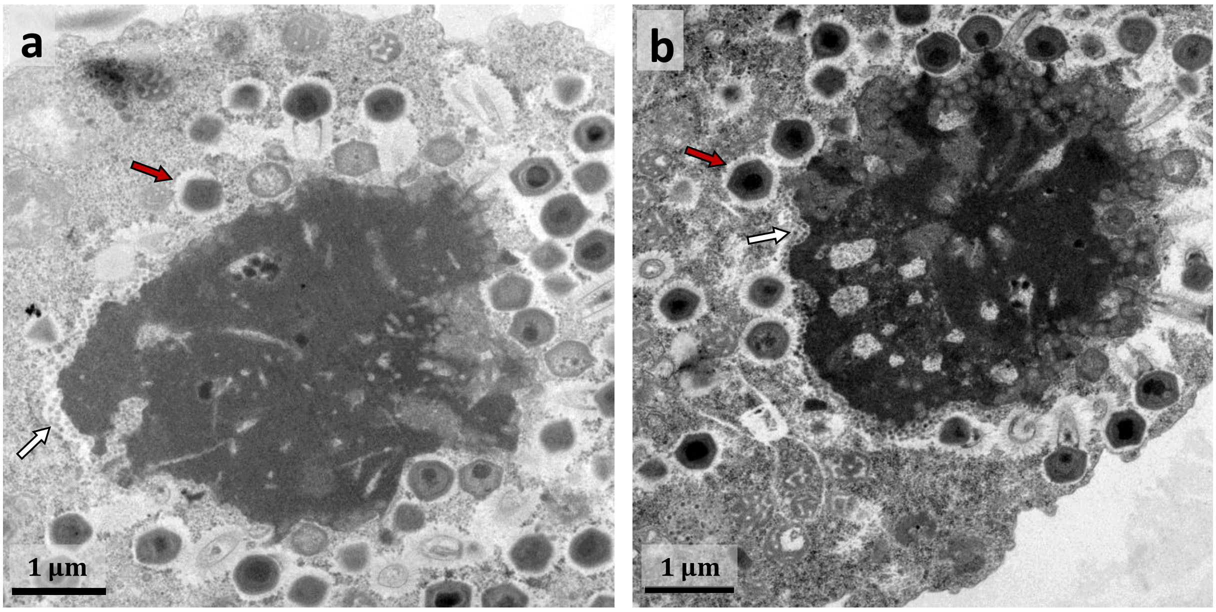

Supplementary Figure 4: Simultaneous production of Tupanvirus and Zamilon virions in coinfected *A. castellanii* cells. (**a** and **b**) *A. castellanii* cells were simultaneously inoculated with Tupanvirus Deep Ocean and Zamilon at MOIs of 10. Zamilon virophage progeny (white arrows), Tupanvirus particles (red arrows).

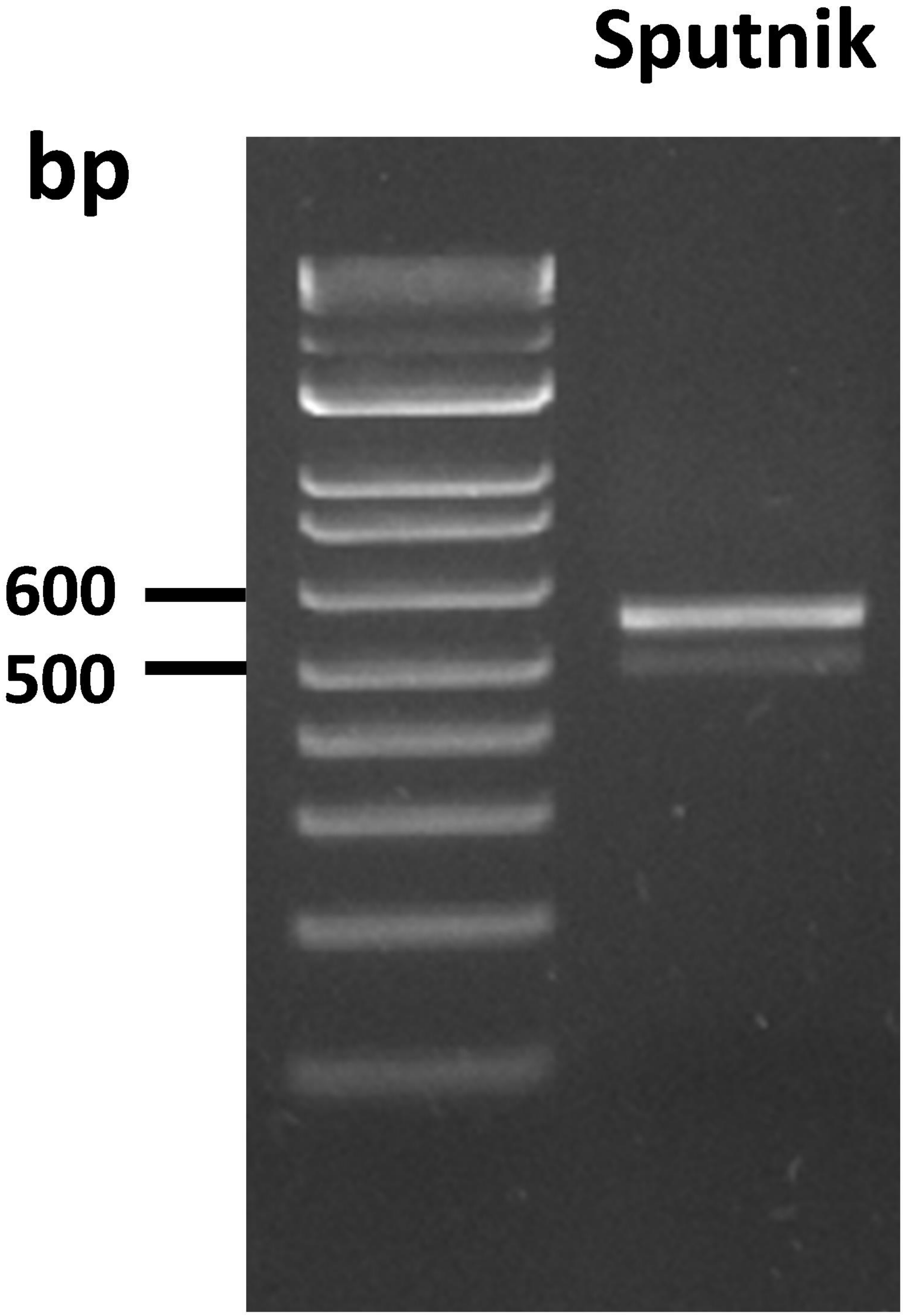

Supplementary Figure 5: Detection of deletion of the 81-nucleotide sequence in Sputnik virophage by PCR. To detect the mutation in Sputnik, the virophage was propagated with APMV and purified as described above. Sputnik DNA was extracted from the purified virophage. Five nanograms of Sputnik DNA were then used as a template for PCR. Sanger

sequencing on each band confirmed the mutation. The primers used for PCR and Sanger sequencing are the same as Guarani.

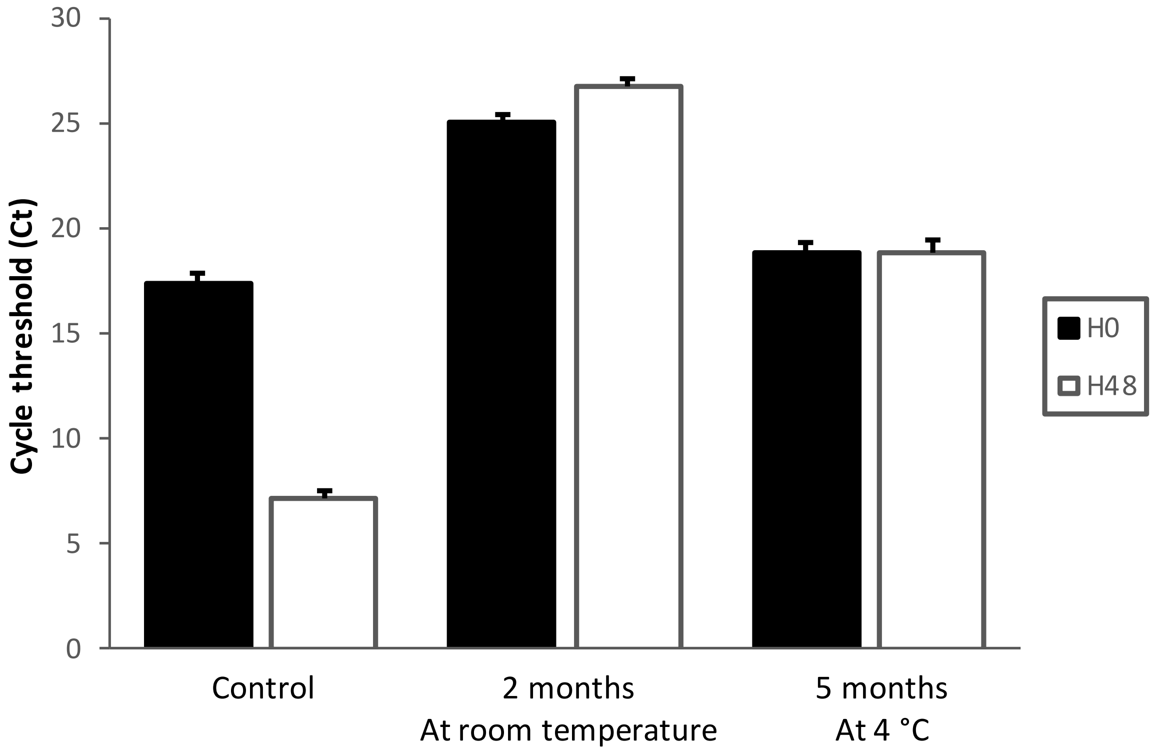

Supplementary Figure 6: Effect of conservation on Guarani virion viability. The virophage was isolated from Tupanvirus supernatant (passage 2) and then incubated at room temperature for 2 months or conserved at 4°C for 5 months, approximatively. Virophage ability to replicate with Tupanvirus was then assessed by real-time PCR as described above. The virophage did not replicate in either condition.

Supplementary discussion (perspectives)

Our study raises some relevant questions that could be addressed in future works. Namely, the capacity of the pure mutant Guarani to replicate and cause the inhibition of Tupanvirus. To this end, we will try to purify mutant Guarani by using end-point dilution coupled with PCR screening to clone the mixture used in this study. Another point that remains to be investigated is how different are Guarani and Sputnik during Tupanvirus infection as the same mutation was detected in Sputnik native population. Last and not least, it would also be interesting to study the evolution of virophage genetic diversity during long-term passage experiments with different mimiviruses from the three phylogenetic clades but also with their distant relatives.
