# Supplementary material for: A virophage cross-species infection through mutant selection represses giant virus propagation, promoting host cell survival
Source: Commun Biol. 2020 May 21;3:248. doi: 10.1038/s42003-020-0970-9 (PMC7242381; doi:10.1038/s42003-020-0970-9)
Supplement: Supplementary file 2 — Description of Additional Supplementary Files [file 42003_2020_970_MOESM2_ESM.pdf]

Description of additional supplementary files

Supplementary data 1: Nucleotide sequences of the collagen-like gene in wild-type and mutant Guarani.

Supplementary data 2: Genome sequences of wild-type and mutant Guarani.

Supplementary data 3: Sanger sequencing products of the collagen-like gene in wild-type and mutant Guarani.

Supplementary data 4: Source data used for graphs shown in figures 1, 3e, 4a, 4c, 6a-6c, 6e, 7 and 8b-8c.
